# Supplementary figures and images for: Exploring the lncRNA localization landscape within the retinal pigment epithelium under normal and stress conditions
Source: BMC Genomics. 2022 Jul 26;23:539. doi: 10.1186/s12864-022-08777-1 (PMC9327364; doi:10.1186/s12864-022-08777-1)

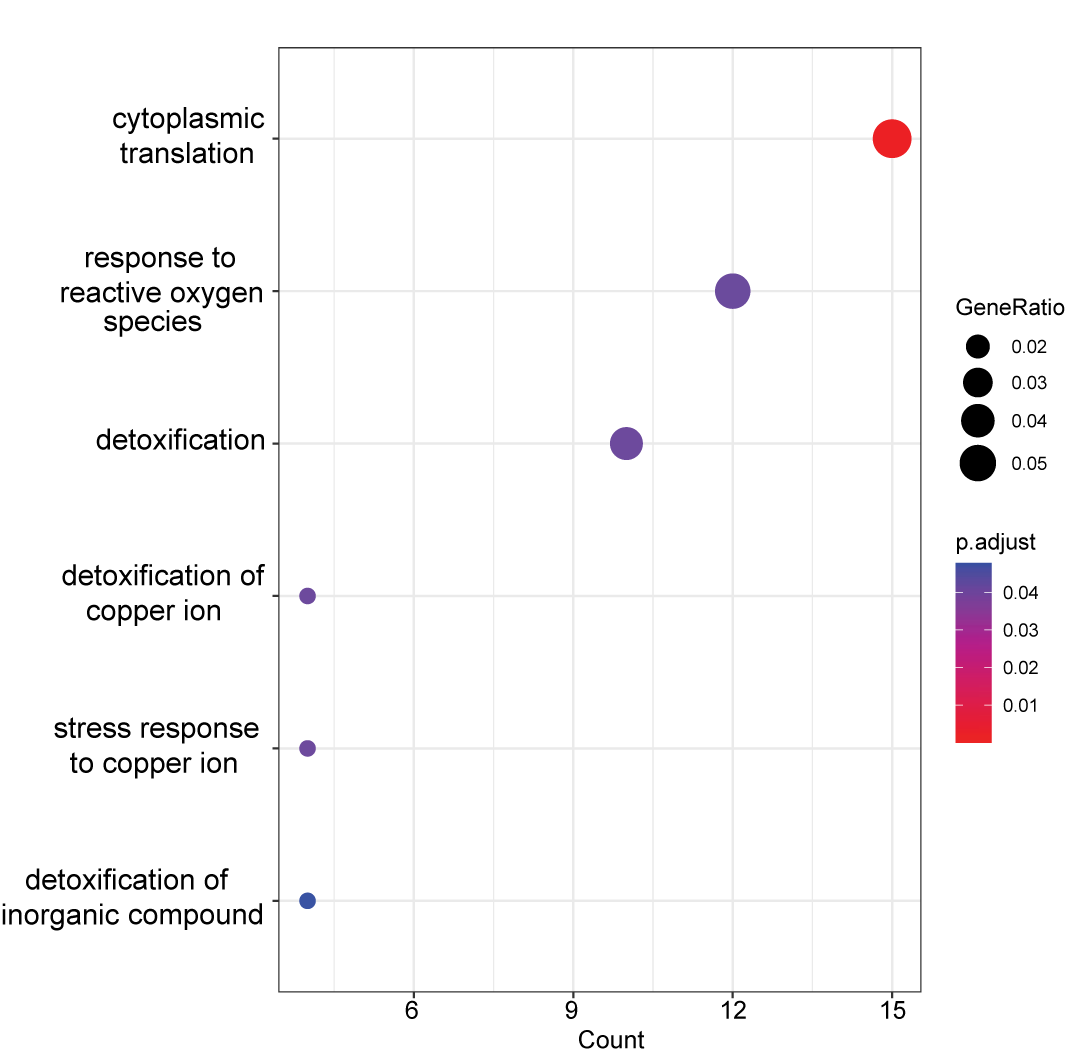

Supplement: Supplementary file 1 — Additional file 1: Supplementary Figure 1. H2O2 treatment of iPSC-RPE leads to upregulation of genes involved in oxidative stress response. Advanced bubble chart shows the GO pathway analysis of genes upregulated in the BXS-H2O2 samples compared to the BXS control samples. The Y-axis label represents the pathway, and the x-axis label represents the number of differentially expressed genes enriched in a pathway. Size and color of the bubble represent gene ratio (gene ratio = amount of differentially expressed genes enriched in the pathway/amount of all genes in the pathway) and enrichment significance, respectively. [file 12864_2022_8777_MOESM1_ESM.tif]

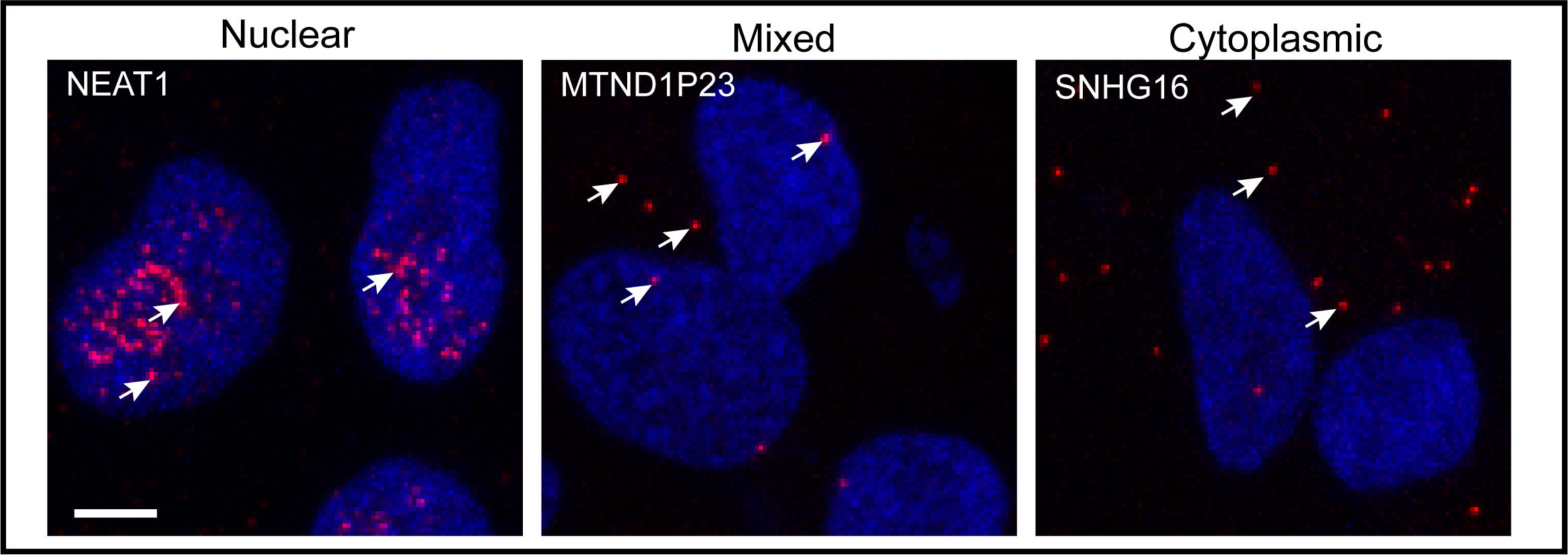

Supplement: Supplementary file 2 — Additional file 2: Supplementary Figure 2. Verification of lncRNA localization in ARPE-19 cells. RNA-FISH images of ARPE-19 cells confirming localization of NEAT1, MTND1P23, and SNHG16 (red) and counterstained with Hoechst solution (blue). Arrows indicate some of the localized RNAs. Scale bar is 5 µm. [file 12864_2022_8777_MOESM2_ESM.tif]

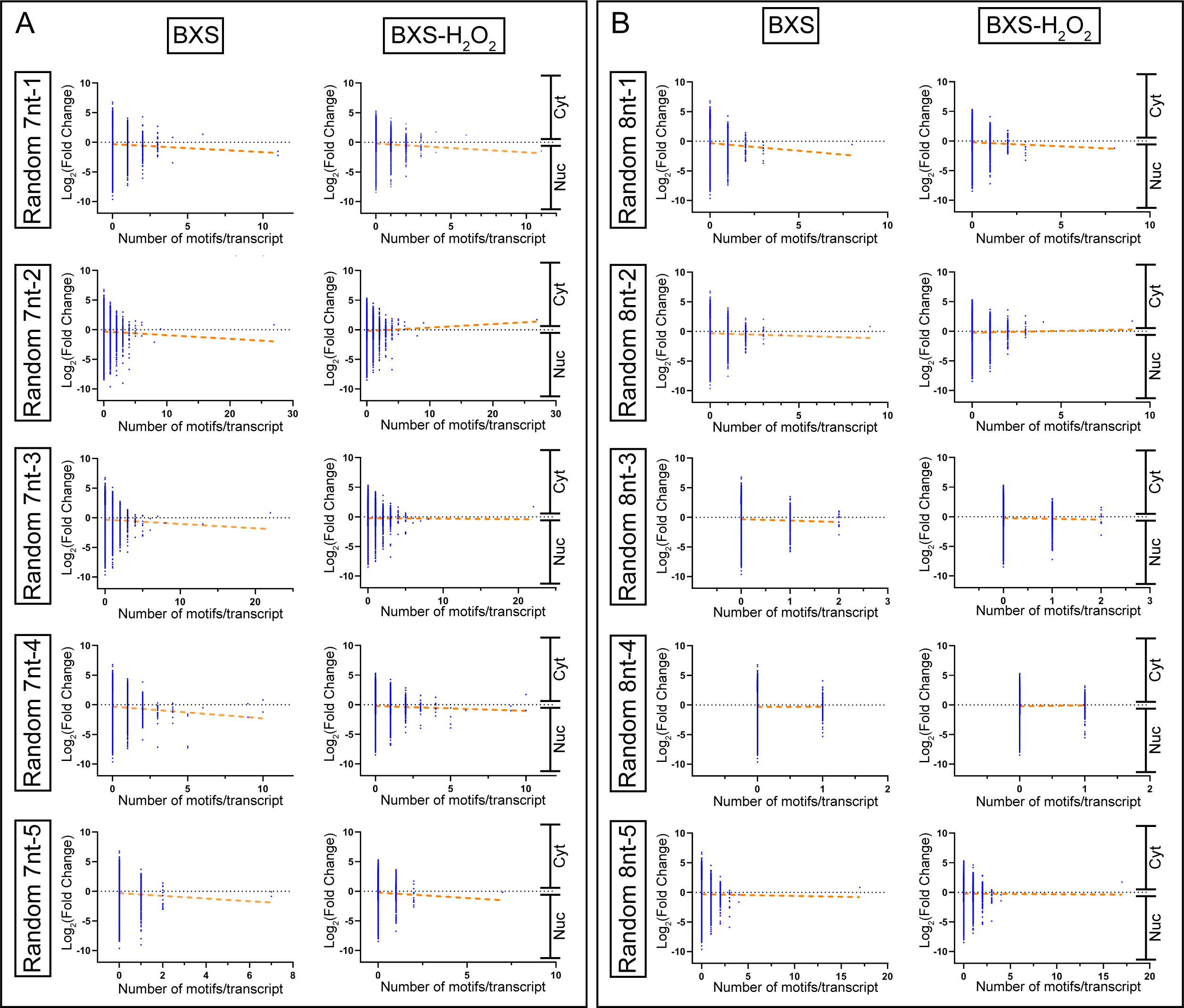

Supplement: Supplementary file 3 — Additional file 3: Supplementary Figure 3. The number of random motifs is not correlated well with localization. Graphs plotting the number of 7-nucleotide (A) and 8-nucleotide (B) motifs per transcript versus log2 cytoplasm:nuclear fold change in both BXS and BXS-H2O2. Fold change corresponding to nuclear (nuc) and cytoplasmic (cyt) localization is indicated. The orange dotted lines plot the trendlines for the data. Five different random motifs of each length were analyzed. [file 12864_2022_8777_MOESM3_ESM.tif]

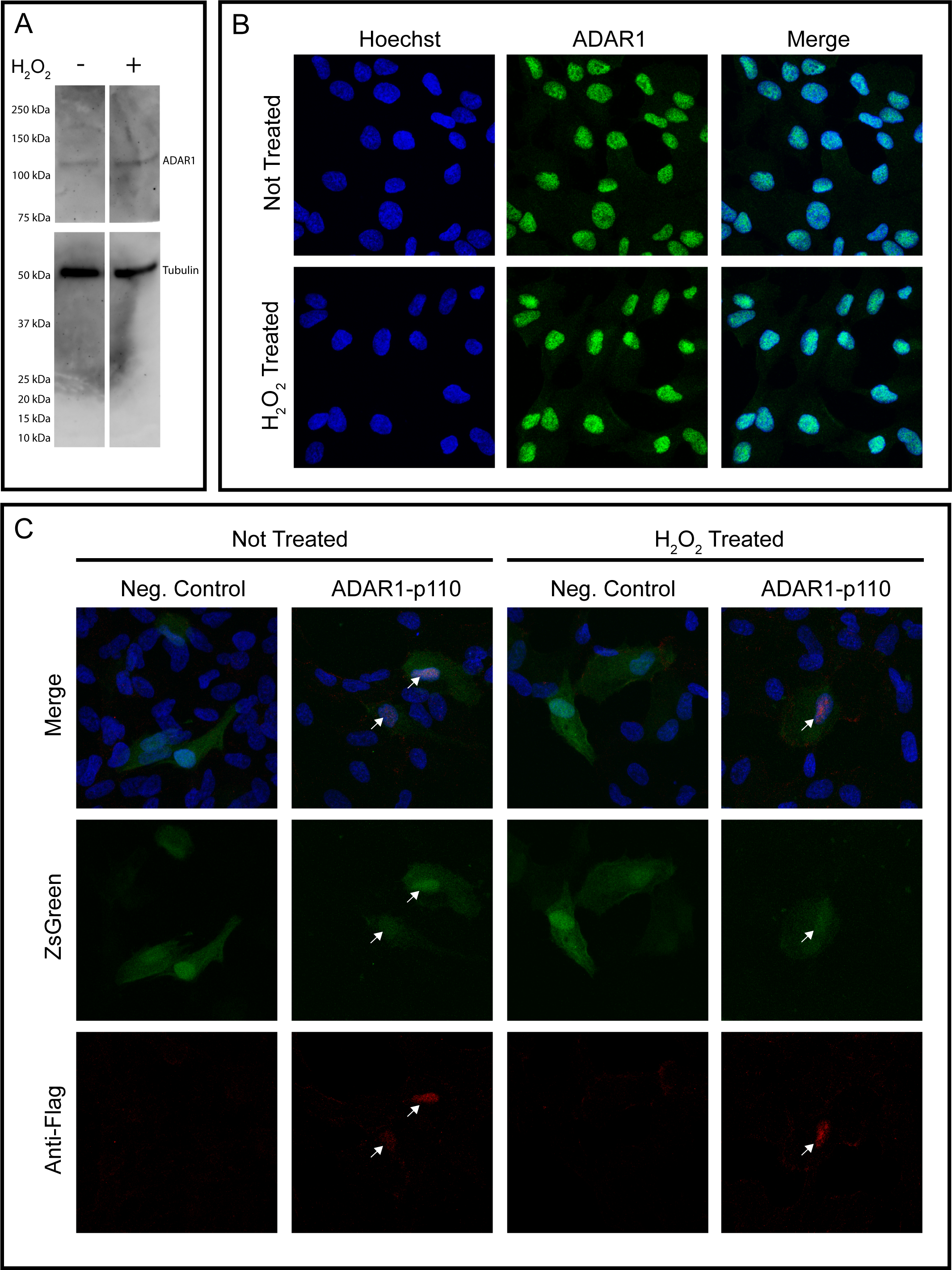

Supplement: Supplementary file 4 — Additional file 4: Supplementary Figure 4. Analysis of ADAR1 expression and localization. (A) Detection of ADAR1 by western blotting of lysates from ARPE-19 cells that were treated with H2O2 or left untreated. Blot images are cropped for clarity and conciseness. (B) Immunofluorescent staining of ADAR1 in ARPE-19 cells that were treated with H2O2 or left untreated. (C) Immunofluorescent detection of ADAR1-p110 in ARPE-19 cells that were treated with H2O2 or left untreated and that were transfected with either only ZsGreen mRNA (Neg. Control) or ZsGreen mRNA plus ADAR1-p110-Flag mRNA (ADAR1-p110). Successful transfection was visualized by fluorescence of the ZsGreen protein, and ADAR1-p110 expression was visualized via an anti-flag antibody. [file 12864_2022_8777_MOESM4_ESM.zip › Figure_S4.tif]

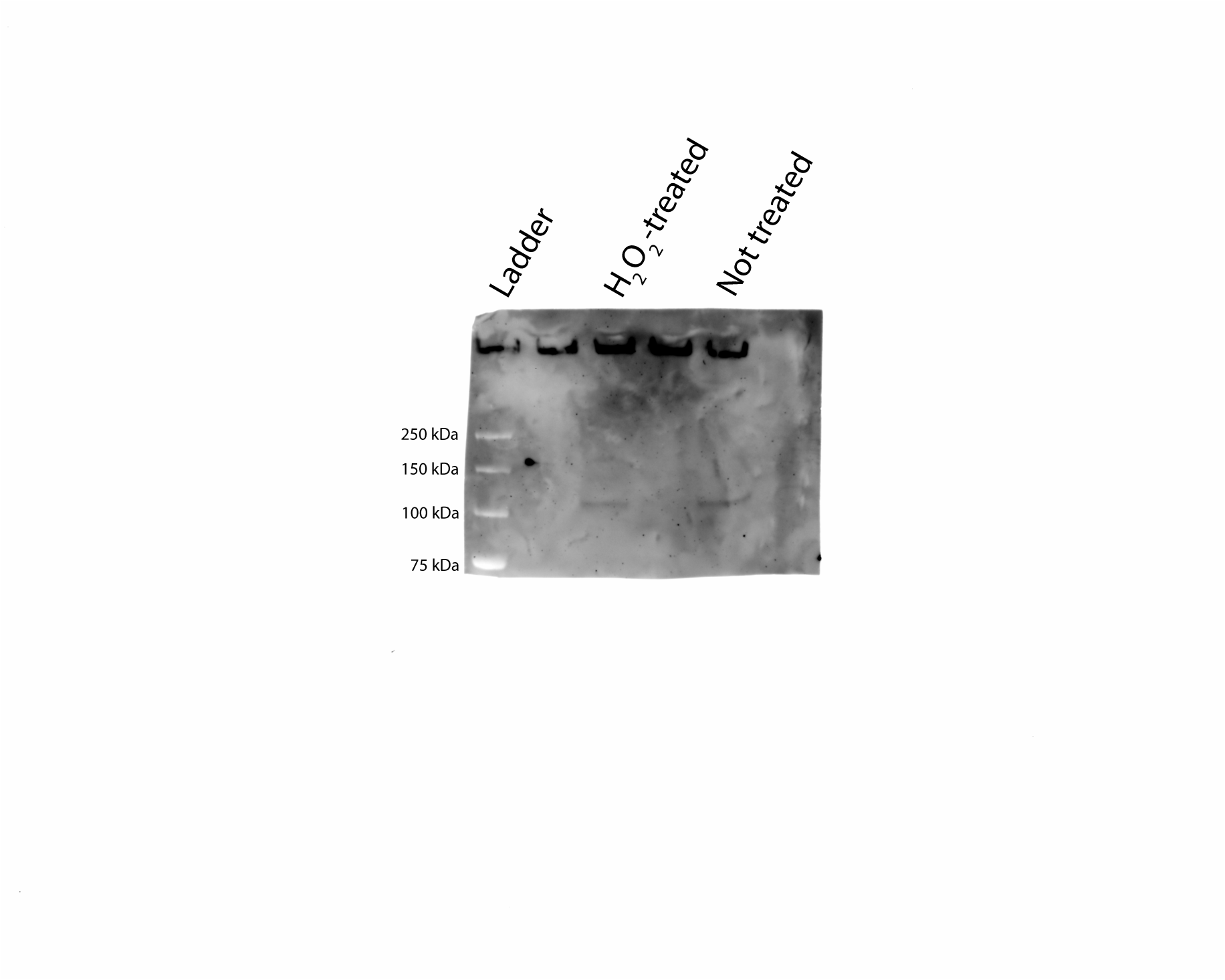

Supplement: Supplementary file 4 — Additional file 4: Supplementary Figure 4. Analysis of ADAR1 expression and localization. (A) Detection of ADAR1 by western blotting of lysates from ARPE-19 cells that were treated with H2O2 or left untreated. Blot images are cropped for clarity and conciseness. (B) Immunofluorescent staining of ADAR1 in ARPE-19 cells that were treated with H2O2 or left untreated. (C) Immunofluorescent detection of ADAR1-p110 in ARPE-19 cells that were treated with H2O2 or left untreated and that were transfected with either only ZsGreen mRNA (Neg. Control) or ZsGreen mRNA plus ADAR1-p110-Flag mRNA (ADAR1-p110). Successful transfection was visualized by fluorescence of the ZsGreen protein, and ADAR1-p110 expression was visualized via an anti-flag antibody. [file 12864_2022_8777_MOESM4_ESM.zip › Figure_S4A_ADAR1_blot.tif]

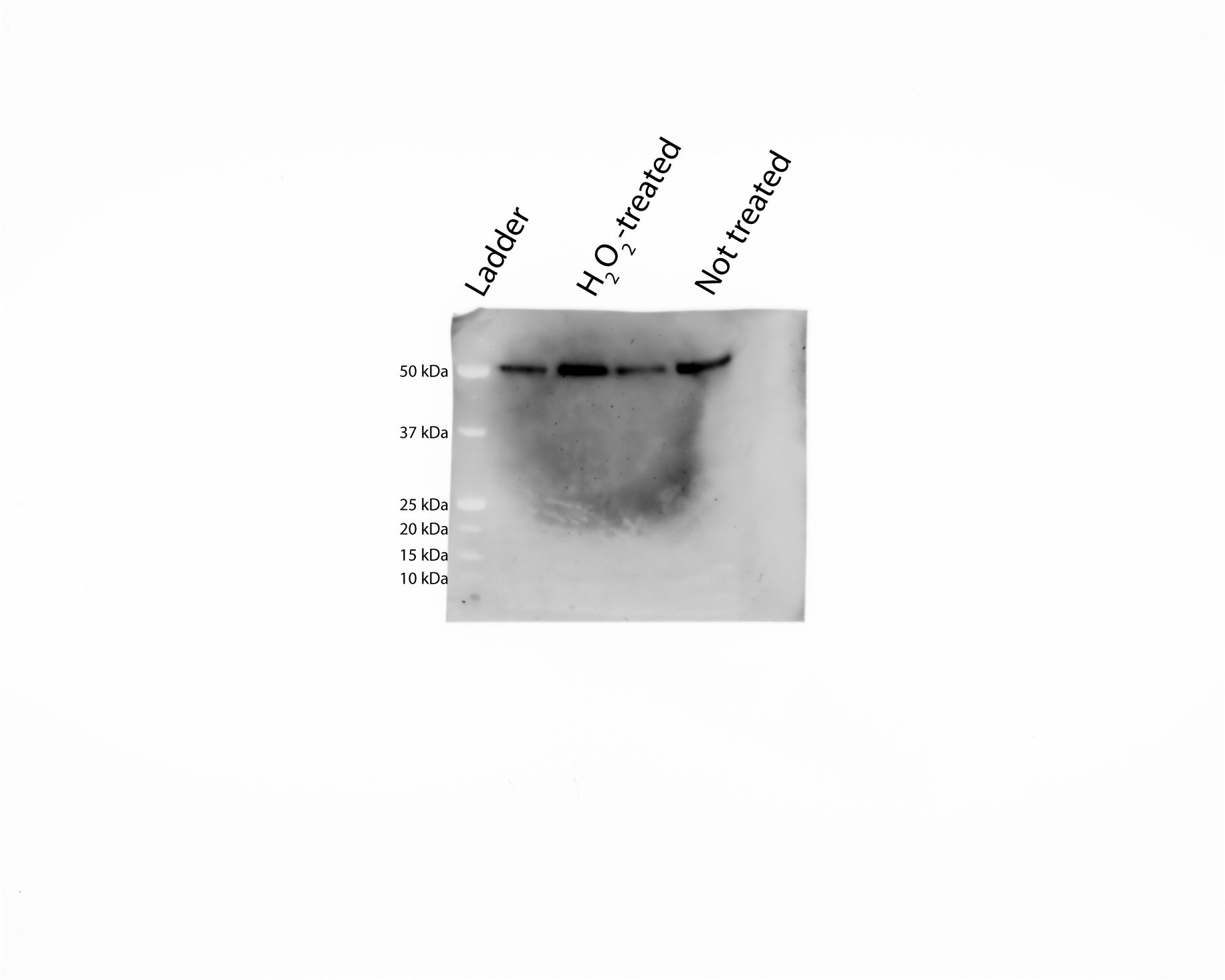

Supplement: Supplementary file 4 — Additional file 4: Supplementary Figure 4. Analysis of ADAR1 expression and localization. (A) Detection of ADAR1 by western blotting of lysates from ARPE-19 cells that were treated with H2O2 or left untreated. Blot images are cropped for clarity and conciseness. (B) Immunofluorescent staining of ADAR1 in ARPE-19 cells that were treated with H2O2 or left untreated. (C) Immunofluorescent detection of ADAR1-p110 in ARPE-19 cells that were treated with H2O2 or left untreated and that were transfected with either only ZsGreen mRNA (Neg. Control) or ZsGreen mRNA plus ADAR1-p110-Flag mRNA (ADAR1-p110). Successful transfection was visualized by fluorescence of the ZsGreen protein, and ADAR1-p110 expression was visualized via an anti-flag antibody. [file 12864_2022_8777_MOESM4_ESM.zip › Figure_S4A_Tubulin_blot.tif]
